# Experiences and wellbeing of family members and carers, regarding PARCS across Victoria

**DOI:** 10.3389/fpsyt.2026.1777053

**Published:** 2026-06-16

**Authors:** Bridget Elizabeth Hamilton, Lisa Mary Brophy, Carol Ann Harvey, Ellie Fossey, Michelle Swann, John Reece, Emma Morrisroe, Victoria J. Palmer, Graham Nicholas Meadows, Cathrine Mihalopoulos, Long Le, Vrinda Edan, Justine Fletcher

**Affiliations:** 1 Centre for Mental Health Nursing, School of Health Sciences, The University of Melbourne, Parkville, VIC, Australia; 2 The ALIVE National Centre for Mental Health Research Translation, The University of Melbourne, Parkville, VIC, Australia; 3 School of Allied Health, Human Services and Sport, La Trobe University, Melbourne, VIC, Australia; 4 Centre for Mental Health and Community Wellbeing, Melbourne School of Population and Global Health, The University of Melbourne, Melbourne, VIC, Australia; 5 Department of Psychiatry, The University of Melbourne, Parkville, VIC, Australia; 6 Department of Occupational Therapy, School of Primary and Allied Health Care, Monash University, Melbourne, VIC, Australia; 7 Department of Health, Government of Victoria, Melbourne, VIC, Australia; 8 Discipline of Psychological Science, ACAP University College, Melbourne, VIC, Australia; 9 School of Public Health and Preventive Medicine, Faculty of Medicine, Nursing and Health Sciences, Monash University, Melbourne, VIC, Australia

**Keywords:** carers, experience, family, recovery, sub-acute care, wellbeing

## Abstract

**Introduction:**

When consumers experience mental health crises, carers are often key supporters and are also impacted significantly themselves. The Prevention and Recovery Care (PARC) model offers a community-based residential program for consumers, in times of mental health crisis. The potential of PARC services to engage carers is under-examined. This study addresses two questions: How do carers experience the PARC service; and what are carers’ experiences of their own wellbeing, during and after engagement with PARC?

**Methods:**

This is a mixed-methods convergent study of carer experience, whereby quantitative survey data and qualitative survey and interview data were gathered, analysed concurrently, and integrated to report carers perspectives of PARC services. Carers reported their wellbeing across 4 timepoints (*n* = 71) and also their experience of PARC services in a Carer Exit Survey (*n* = 50). An independent sample of six family members, each from a different PARC service, engaged in semi-structured telephone interviews.

**Results:**

For service experience, carers rated the PARC service as highly satisfactory. Interviewees reported a sense of relief, gratitude, and period of regrouping, while valuing the PARC service and feeling positive about accessing PARC services in the future. Positive experience was defined in contrast with distressing experiences of acute wards; concerns were expressed about limits to timely access of PARC services in future if needed. Regarding carer wellbeing, time 1 levels varied across participants, and all measures showed improvement for carers over time. They reported experience of respite, with confidence to entrust their family member to the team, and learning from PARC service staff ways to cope and interact with their family member.

**Discussion:**

Carers considered the PARC service a positive environment for the person to receive treatment and support and also experienced PARC services as supporting their own quality of life and wellbeing. This study contributes evidence about how highly valued these recovery oriented sub acute residential services are for carer service users; however, there is potential to further enhance the engagement of carers in PARC service delivery, including through inclusion of carers in co-design.

## Background

Most people with direct experience of mental health challenges and service use (referred to here as consumers) have ongoing and mutually supportive relationships with family members (1). Support provided by families to consumers may include: emotional support, practical assistance, financial support and advocacy and assistance to access healthcare services (2, 3). Families also provide relational support and facilitate connections for loved ones within communities. Depending on age and stage, and life circumstances, families may engage more in times of crisis or when a consumer is experiencing poorer health (4), or they may be the mainstay of continuous support for people.

The impacts of mental health issues are experienced by families (the term here may refer to partners of people, children and siblings, wider extended family members and kin) and significant others as well as consumers. Positive impacts experienced by families include enjoying satisfying relationships with the consumer and personal skill development (2). Negative impacts include: distressing emotional responses, ranging from grief and loss to guilt and self-blame; disrupted family relationships; and reduced employment and financial hardship (3, 5–7). Family members also experience depression and anxiety, social isolation, physical health impacts and decreased quality of life (2, 8–10).

Many family members want to be involved in finding the right assistance and in supporting recovery of their relative living with mental ill-health, and this is also desired by most consumers (5, 11, 12). Family members and carers often seek information about the diagnosis of the person they care about and the impact of illness, as well as how to navigate services and become involved in care planning (1, 13, 14). Families also need their own support, including: to discuss their experiences; improve family relationships; access financial assistance; and to consider alternate caring arrangements (1, 13, 14). This includes that children within families in particular may need greater attention in care models and support provision.

The focus of this paper is the experience and needs of family members, supporters and kin who are engaged with a consumer during a period of crisis occasioning a PARC service admission. While acknowledging the diversity of roles and preferred descriptors also evident across literature, here we mainly use the term ‘carer’. New models of care often express intention to improve both consumer and carer engagement and experience, including maximising choice and consent. Internationally, residential alternatives to hospitalization in psychiatric inpatient units, such as the Australian Prevention and Recovery Care (PARC) services based in Victoria (15, 16), are increasingly available across Australia and may be preferred by both consumers and carers, as they aspire to provide a less restrictive and more home-like alternative (17–19). They may also enable opportunities for more recovery-oriented service delivery (20) and residential rehabilitation (12).

PARC services are operated as a partnership between nongovernment agencies (known as Mental Health Community Support Services (MHCSS) in Victoria working across States and Territories) and local clinical mental health services. Short-term treatment and care are offered for up to 28 days, to support people with severe mental ill-health to either avoid a psychiatric hospital admission (step-up care) or transition from hospital back into the community (step-down care). These services have potential to provide needed support to families as well as consumers (15). According to the Victorian government’s PARC services framework and operational guidelines (21), potential benefits to families of the service model include decreasing “the possible difficulties and stresses experienced by carers, in supporting people who are acutely unwell and are receiving community treatment.” (Department of Health, page 10). Possibilities for early intervention in crises are particularly emphasised. PARC services could be useful for encouraging links to consumers’ natural supports, including family and friends. The guidelines encourage collaborative therapeutic relationships with families and carers, carer participation in planning, delivery and evaluation of adult PARC services, and a facility which is welcoming of families and responsive to consumers’ diverse family circumstances (21).

Existing knowledge is limited about the appropriateness and effectiveness of PARC services and their place within the wider mental health system, especially in relation to families (22, 23). However, a mixed-methods evaluation of a Victorian PARC service for young people indicated that families viewed the service as filling a gap on the continuum between community services and acute inpatient wards, consistent with their intended purpose (24). Findings also suggested that families felt included in service delivery, valued the service’s recovery-oriented practice (such as promotion of their relative’s autonomy and self-help), and found the youth PARC service environment safe and welcoming (24). However, negotiating the appropriate level of involvement of family in care (e.g., in decision making) can be challenging (25), as also reported in an evaluation of an adult PARC service in Queensland (22). It has been found in previous work by this team that engagement with families varies across PARC services in Victoria (15), specifically in regard to how they were included in care meetings. This study explored the experiences of family members and carers of consumers who have used PARC services across Victoria. The research questions addressed were:

- How did family members, carers and supporters experience the PARC service?
- What are carers’ experiences of their own wellbeing, during and after engagement with PARC?

## Methods

### Study design

This was a mixed-methods longitudinal convergent triangulation study of carer experience, nested within a longitudinal cohort study of Victorian PARC services (26). A detailed description of the overarching study design, recruitment and data collection can be found in Brophy et al. (26) but in brief this was an analysis of the experience and trajectory of self-reported personal recovery and other outcomes, for consumers from Victorian PARC services, using data collected over 4 time points from admission to 12 months post discharge. This carer sub-study employs a convergent triangulation design (27) whereby quantitative and qualitative data were gathered and analysed separately and then integrated, to strengthen findings regarding carers’ perspectives of PARC services. The impact on carers’ quality of life and wellbeing were explored via a longitudinal survey (see Table one) and that approach was complemented by a satisfaction survey and qualitative interviews conducted soon after an episode of PARC service for a consumer. Interviews were included to elicit rich accounts of carer experiences, beyond what is commonly gathered through collection of carer satisfaction data. Both survey and interview samples were stratified, to include perspectives from carers across as many Victorian PARC services as possible.

### Setting

In the research period, 20 adult PARC services were offering 194 beds and some day-visit only places in Victoria, Australia. Nineteen of the 20 PARC services were sub-acute, offering a 28-day maximum stay, while the remaining service offered an extended stay of 6 months (26). One of the 19 sub-acute PARC services was a woman-only service and the others were mixed gender environments. All 19 adult sub-acute PARC services were included in this study, but the extended-stay PARC service was excluded. Of these PARC services, three were located in inner-city (metropolitan) areas, 12 in suburban areas, and the remaining four in regional areas. At the time of writing, 19 adult PARC services were in operation in Victoria.

The staffing profiles of PARC services varied, but may have included Occupational Therapists, Social Workers, Psychologists, Peer workers, Art Therapists and Mental Health Support workers. PARC consumers movements in and out of the PARC were not restricted. Whilst staying in a PARC services consumers had access to a range of group programs focused on some of the following areas, recovery and wellness, psychotherapeutic interventions, physical health and activities of daily living.

### Recruitment and data collection

Recruitment of consumer participants was conducted by a team of consumer and non-consumer researchers who visited PARC services and invited eligible residents to participate. Consumers were also informed about the carer arm of the study and were invited to nominate a carer contact who may want to be involved. A carer researcher contacted each nominated carer by phone, email or post and invited them to participate in the study. If the carer agreed to participate, they were offered options of a survey online or over the phone, or via post and were provided with a $25 supermarket gift voucher for their time. Two weeks following the consumer participant’s exit from the PARC service, carer participants were contacted again to complete a second set of questionnaires and received a $10 supermarket gift voucher. This process was repeated at 6 and 12 months following the consumer participants PARC service exit date; see **Table 1** for timing of measure completion.

**Table 1:** Description of measures and timing.

Measure	Reference	Domain	Description	T1	T2	T3	T4
				one-week after entry	within one-week of exit	six-months after exit	12-months after exit
Assessment of Quality of Life – 8D (AQoL8D)	(28)	Quality of Life	The AQoL8D is a 35-item quality of life measure with eight dimensions: independent living, relationships, mental health, coping, pain, senses, self-worth, and happiness. Utility scores derived from the AQoL8D algorithm range from 1.00 (representing perfect health or best possible health on that questionnaire), to a negative score of − 0.04 (representing the worst possible health state that has been valued as worse than death). The Australian utility scoring algorithm was used.	X	X	X	X
Kessler 10 (K10)	(29)	Psychological distress	The K10 consists of 10 items measured on a five-point Likert scale and is scored by adding all responses. Possible scores are between 10-50, with higher scores indicating greater distress	X	X	X	X
Warwick Edinburgh Mental Wellbeing Scale (WEMWBS)	(30)	Wellbeing	The WEMWBS consists of 14 items measured on a five-point Likert scale with a total score calculated by adding all responses. Possible scores are between 14-70, with a higher score indicating greater wellbeing.	X	X	X	X
Carer Exit or Satisfaction Survey	(31)	Satisfaction	The Carer Exit or Satisfaction Survey is a 13-item self-report questionnaire developed by Mind Australia to assess consumer satisfaction of mental health services. It is a bespoke instrument based on the Mind Australia Satisfaction Survey that was initially based on a similar survey developed by Rethink in the UK. It includes 10 quantitative questions with responses on a four-point Likert scale with a fifth option to decline to respond. The possible score ranges from 0-40, with a higher score indicating greater satisfaction. The remaining three items are open-ended questions asking “What has been the most helpful thing about the PARC service for your family?”; “If you could change anything about the service what would it be?”; and “Any other comments and feedback?”.				X

Recruitment for the semi-structured interviews occurred via flyers posted at each PARC service and by contacting individuals who expressed interest in participating in the longitudinal study who were ineligible, as quotas were filled. Only one interview participant from each PARC service was sought, and all family members and carers were recruited with the knowledge and permission of the consumer. Interested parties contacted the research team and interviews were conducted via phone. All Interviews were conducted by a carer researcher and lasted for between 15 and 35 minutes. These were audio-recorded, transcribed and deidentified; all data were stored securely.

### Measures

Quality of life was chosen as an important outcome measure to determine the impact of a PARC service stay on carers, and whether any benefit from a PARC service stay for the consumer translated to improved carer quality of life. The quantitative wellbeing outcome measure used in this study was the Assessment of Quality of Life (AQoL)-8D (28). Other quantitative outcome measures were the Kessler Psychological Distress Scale (K10) (29), Warwick Edinburgh Mental Well-Being Scale (WEMWBS (30) and the Carer Exit Survey, a bespoke instrument previously developed by Mind Australia to gauge carer satisfaction with the service (31). **Table 1** summarises all measures.

### Data analysis

Quantitative data were analysed using SPSS 26. Following data cleaning and assumption testing, data across the four time periods were analysed using longitudinal growth curve analysis via the SPSS linear mixed model procedure. Change trajectories were analysed for linear, quadratic, and cubic trends. Effect sizes measures with associated 95% confidence intervals are provided for all inferential outcomes.

#### Growth curve analysis

The growth curves for the five measures recorded at four time periods were analysed using linear mixed modelling. All models were fitted with random slopes and intercepts. A range of covariance structures were evaluated for both the random effects and the repeated measures. A diagonal covariance matrix for the random effects combined with a scaled identity matrix for the repeated effects resulted in all models converging satisfactorily along with adequate model fit according to the AIC criterion. Full information maximum likelihood estimation was used for all models, and assumption testing found that data were missing at random (MAR). Linear, quadratic, and cubic growth trajectories were evaluated.

#### Carer exit survey

The Likert scale responses in the carer exit survey were analysed descriptively (frequencies and percentages).

#### Quantitative data preparation and assumption testing

Data for quantitative outcomes were entered into a single SPSS 26 data file for analysis.

Although data were available from a potential pool of 71 participants, missing data levels were high, ranging from 1.4% (WEMWBS at time 1) to 49.3% (K10 at two weeks). As might be expected, missing data levels increased across the duration of the study with a mean of 43.7% missing data across the five outcome measures at 12 months (time 4) versus 17.4% at time 1. Generally, missing values were highest for the K10 (*M* = 40.9% across the four time periods) and lowest for the WEMWBS (*M* = 25.1% across the four time periods). Despite assumption testing showing that data were missing at random, the level of missing data precluded any form of data imputation, so data were analysed as available, which should be considered when evaluating the findings. In support of this decision, simulation testing using a hypothetical imputed data set revealed no notable difference in the pattern of outcomes.

Power considerations were based on a single-group within-subjects design measured over four periods. The lowest sample size (*n* = 36) had adequate power (.8) to detect at least medium effects (*f* = 0.25), with the average sample size across the four time periods (*n* = 55) having adequate power to detect small effects (*f* = 0.1). Large effects had very high power for being reliably identified (>.95). Hence, the study was seen to be adequately powered.

Some level of skew was evident for all outcomes, but not to the extent of compromising the outcomes of the desired analyses. Further, a range of transformations failed to notably improve the distribution of scores, so the data were analysed in their original form. All other assumptions for the reported analyses were adequately met.

#### Qualitative data analysis

The semi-structured interview transcripts (*n* = 6) and the qualitative responses from the Carer Exit Survey (n=*50*), consisting of 230 individual free text responses ranging from single word to 100 word responses, were analysed thematically by two researchers (BH and JF). Sub-themes and themes were developed inductively during data analysis (32). As the analysis team did not conduct the interviews, the audio files of the interviews were listened to several times while reading transcripts. Likewise, the collated free text responses were read and re-read by both analysts for data immersion in preparation for coding. Coding was undertaken by the primary qualitative researcher BH; a subset of data was coded independently by CH and only minor differences of emphasis were found. The matrix of data, coding and thematic structure was discussed in detail until agreement was reached across 4 authors: BG CH JF and LB. As qualitative data was generated from both the survey and the semi-structured interviews and analysed together, the quotations reported here are identified as arising from an interview or a survey. Quantitative and qualitative findings were integrated for explanatory value in the process of reporting (27).

#### Mixed methods integration

Qualitative answers from survey participant were combined with interview transcript data, for qualitative coding and thematic analysis. Then all authors met and compared in detail the quantitative and qualitative data analyses, observing contrasting and confirming intersections and then developing cross cutting points for discussion. For triangulation we considered: How do qualitative themes and subthemes relate to quantitative findings? How do they provide explanatory detail for the quantitative findings?

## Results

Carers rated their PARC service experience and qualitatively described their encounters with PARC services and staff, including the environments, programs, personal impact, and their contact with consumers. Carers also rated their own quality of life, wellbeing, and distress through the time of the PARC service stay. After reporting the study samples for surveys and interviews and descriptive statistics for the survey data, the qualitative and quantitative findings are presented in two integrated findings sections; *Part 1:* C*arer experiences with PARC services* and *Part 2: wellbeing of carers.*

### Sample

Via surveys, carers (*n* = 71) reported details relevant to their wellbeing in the K10, WEMWBS and AQOL (at two-4 timepoints). From that sample 53/71 carers completed the Carer Exit Survey (T2). Only 35 respondents provided data for all four time points, and 47 respondents provided data for at least three time points. These calculations account for non-consecutive time points: for example, an individual with data at two time points may have contributed responses for time 1 and time 3, but not time 2. An independent sample of carers, each from a different PARC service (n=6 in total) engaged in the semi-structured telephone interviews. The aim was for a larger sample of upto 22 carers, being one from each PARC. However, recruitment relied on two steps of carers opting in after gaining consumer consent and this proved to be challenging, while people were navigating family crisis. **Table 2** presents the sociodemographic data for all survey participants at time 1 and then for the interview participants (Note that not all demographic questions were asked of interviewees).

**Table 2:** Socio demographics of participants.

Demographics	Carer survey participants (*n* = 71)		Carer interview participants (*n* = 6)
	n	%	
Gender			
Male	18	25.4	1
Female	52	73.2	5
Missing	1	1.4	
Country of Birth			
Not Australia	11	15.5	
Australia	60	84.5	
Missing	0	0	6
Children			
Yes	55	77.5	3
No	15	21.1	1
Missing	1	1.4	2
Highest Level of Education			
High school or less	37	52.1	
Diploma, certificate, or university	33	46.5	
Missing	1	1.4	6
Marital Status			
Single	14	19.7	
Married or de facto	40	56.3	4
Separated, divorced, or widowed	16	22.5	
Missing	1	1.4	2
Age			
<20 years	3	4.2	
20–31 years	4	5.6	
32–41 years	6	8.5	
42–50 years	8	11.3	
51–65 years	33	46.5	
66+ years	16	22.5	
Missing	1	1.4	6
Relationship to Consumer Participant			
Partner or spouse	15	21.1	0
Daughter or son	20	28.2	0
Father or mother	17	23.9	3
Brother or sister	10	14.1	3
Friend or room/housemate	4	5.6	0
Other	4	5.6	0
Missing	1	1.4	

Findings are presented in two sections, bringing together qualitative and quantitative data to address each research question in turn.

### Part 1: family carer experiences with PARC services

This section of analysis answers the question: *How did family members experience the PARC services?* The context for qualitative themes from interviews and surveys is set by first presenting results from the Exit survey, reporting carers’ overall satisfaction with PARC services.

#### Exit survey

Fifty-three participants completed the satisfaction (exit) survey, representing all 19 PARC services involved in the evaluation. Each service was represented by between one and five carers. Carer participants supported people with various relationships to themselves; most were reporting about parents or partners (see **Table 2**.). Three respondents were carers with granddaughters and an ex-partner.

**Figure 1:**
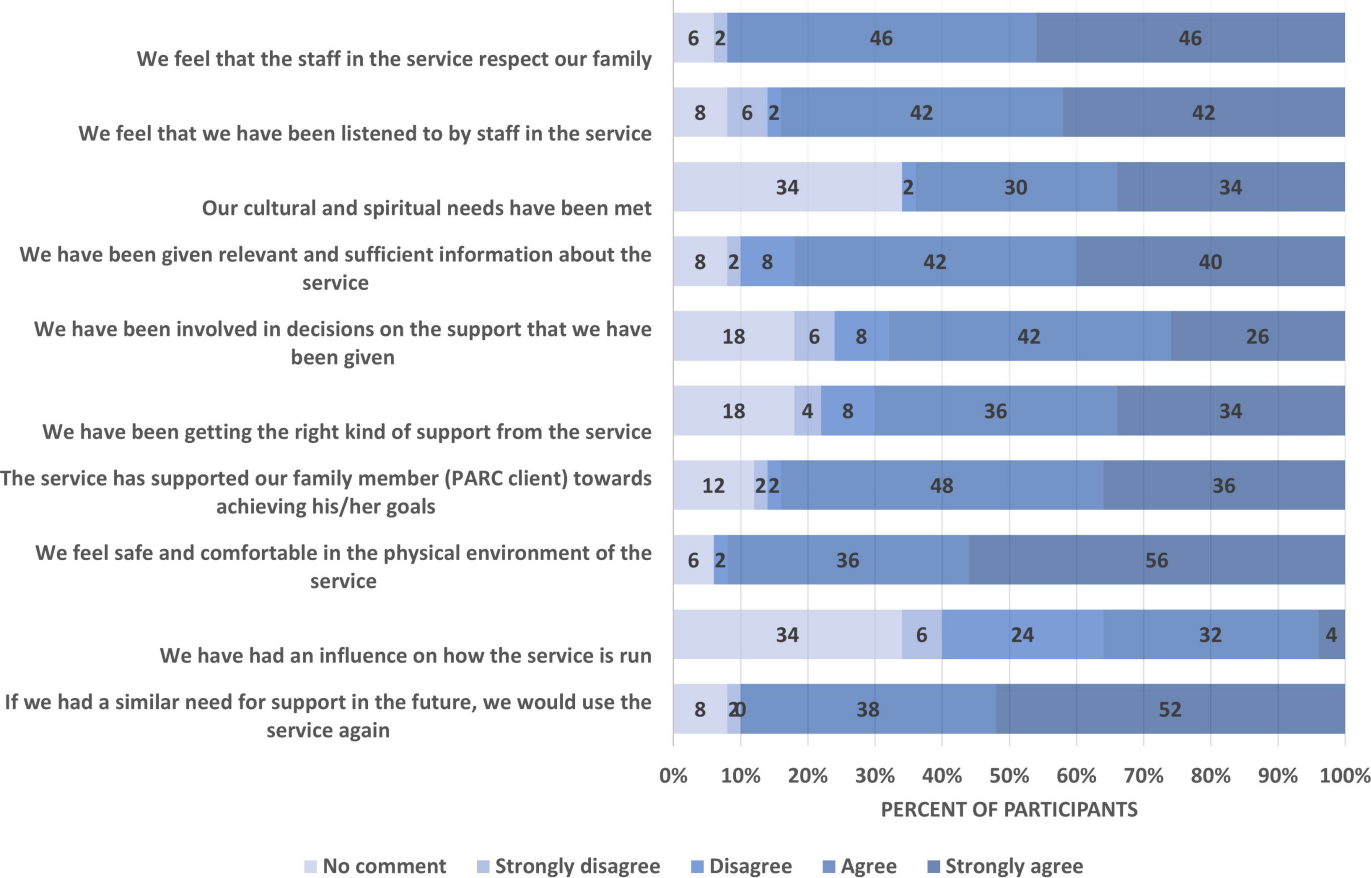
Exit survey (n=53).

The Likert scale response percentages are displayed in **Figure 1** below. The majority (92%) of participants agreed or strongly agreed that the service respected their family, that relevant and sufficient information was provided, and that they were listened to by staff. Participants agreed that the PARC service had supported their loved one towards achieving their own goals, that they felt safe and comfortable at the PARC service, and would use the PARC service again if they had a similar need for support in the future. The majority of participants reported they received the right kind of support from the PARC service.

Participants were generally positive about their involvement in deciding on the support they were given. In terms of the families’ cultural and spiritual needs being met, participants responses were equally divided between no comment (34%), agree (30%) and strongly agree (34%). There was greatest variation in participants’ views about their influence over how the PARC service was run, with 34% with no comment, 32% agreed and 24% disagreed.

Overall, family members rated the PARC service as highly satisfactory. In addition to the quantitative responses, 53 family members provided qualitative comments across three questions in their surveys, related to every one of the Victorian PARC services. In response to the question *What has been the most helpful thing about the PARC service for your family*?, 50 participants commented. Likewise in response to the question *If you could change one thing about the PARC what would it be*? 17 out of 53 participants said they would not change anything, four did not comment, and 34 participants commented. When given the opportunity to provide *any other feedback*, 27 participants made comments, 26 did not.

Thematic analysis of interviews and qualitative survey responses generated two overarching themes, *PARC services work for families* and *PARC service limitations*, and within these were six sub-themes. These were: *Relief from crisis; Supportive and safe; PARC service is better than hospital; Recovery through learning & relationships, Limited access to PARC services; Limited family involvement.* Supporting quotations from both interviews and surveys are provided in **Table 3**.

**Table 3:** Carer experience of PARC services- thematic structure and exemplar quotes.

Themes	Subthemes	Quotes
*PARC services works for families*	*Relief for all from crisis*	it’s very nice to know that he’s being looked after, that he’s in care with somebody 24/7 so that’s very helpful and a big relief to us his family who can’t be checking on him constantly (Interview)
		We appreciate the help that my brother has been given - we would call it a life saver. We really appreciate it (Survey)
	*Supportive and safe*	I cannot praise PARC enough, it has been the sole thing that has helped my son the most of every other type of assistance he has ever had, the staff have an incredible supportive compassionate manner and the staff listen to the client (my son) and are open to helping the carer (myself) and to work together on whatever the issues are specific to the situation (Survey)
		I found it extremely supportive and made everyone feel welcome. They treated everyone there so well - I was amazed (Survey)
	*Better than hospital*	“There’s been several very unhelpful incidences in the emergency ward at [Hospital] – but I can’t think … that we’ve ever had any issues at PARC”(Interview)
		Offering [daughter] a safe place to prevent in most cases hospitalisation because she just gets so fearful of hospitals and won’t go and this just gives her a safe place and respite. Hospital doesn’t help her at all. She gets worse in fact (Survey)
		“The first time we got her into recovery without PARCS, didn’t even know about it, at that stage she was backwards and forwards into the acute ward, …I can’t emphasise enough how much PARCS is important for us” (Interview)
	*Recovery through* *relationships*	Support and accountability for our son to follow expectation to rebuild himself (Survey) When you are dealing with the same people more often, I guess you’re able to develop more of a relationship with them, I think brings about more learning really (Interview) I see a major change in the positive direction of his recovery and learning to function in society and at home (Survey)
*PARC service limitations*	*Limited access*	The only thing I would say is she definitely wasn’t ready to come home, but believe me I understand that these places are so few and far between that it’s unfortunate that there are people that they consider more critical, she still wasn’t well (Interview) Took a long time to get help. Took forever to get him in there. He had to wait about 8 weeks. Was in a really bad way before he went in. (Survey)
	*Limited family involvement*	I am not sure if there is an actual care plan or how that works while he’s there (Interview) [I would like] For them to potentially be a bit more inclusive of me [carer] and to let me know a bit more of what is going on with him. Then I will be able to support him along the way. …I would also like to know more so that I can be more of a resource for him, as I am his main support. I would like to know the strategy they have for him after he leaves so I can encourage him in that. I don’t know the plan or what to do when he is having a down moment. I would like to continue on the path that they have set for him. (Survey) They don’t listen to the person that’s actually dealing with the consumer every day. They just take on board what the consumer does and they don’t take notice of what other people say (Survey).

#### Relief for all from crisis

Carers placed their recent PARC service experiences in the context of an intense crisis being experienced by the family, with a lead up of many months. They reported heightened stress and distress between family members, in the time leading up to a PARC service stay and then a very significant sense of relief for all family members when the family member entered the PARC service. After the point of PARC service exit, when engaged in surveys and interviews, carers were processing their experiences and regrouping, with heightened emotions.

#### Supportive and safe

Many carers were effusive in praising the staff and the care provided at PARC services. The sense of safety and confidence in PARC services was expressed by a diverse range of carers and in relation to a wide range of consumers, young and older, men and women, with such comments as: *The security it has given our son, in a caring environment; I felt the staff were tremendous and supportive to my daughter and myself;* and *That my wife was in a safe and helpful environment* (Surveys). Many carers also appreciated the physical environment of PARC services, for being homelike, well cared for and welcoming: *The units were very nice - I could live in one of them!* (Survey). Not every participant experienced their PARC service setting so positively: *Perhaps [PARC needs] a facelift to building* (Survey). Except for three less satisfied comments, carers were overwhelmingly positive about trusting PARC service if needed in the future.

#### PARC service is “better than hospital”

For most carers, the experience of PARC services commonly occurred in the context considerable experience with a full range of mental health services. This was apparent in the qualitative survey comments, when participants placed their recent experience of a PARC service in comparison with other experiences of service use. For example: *[PARC is] The sole thing that has helped my son the most, of every other type of assistance* (Survey).

Most carers emphasised the value of PARC services to their family, in contrast with challenging experiences of acute inpatient wards, details of which they also recounted. Hospital admissions were spoken of as highly problematic for the family member, stigmatised, frightening, unsafe and a situation to be avoided. Acute admission was considered regrettable, even if necessary or the only option at crisis points. In contrast, an episode of care at PARC services if a crisis arose was highly acceptable to carers, and consumers, safe and constructive. Carers recognised the place of PARC services in enabling transitions, especially as a step-down from hospital. They contrasted perceptions of the physical spaces of hospital wards with the quiet and homelike PARC services. The warmth and gratitude carers expressed regarding PARC services contrasted with the mixed emotions they experienced when reflecting on admissions to hospital. We underscore that comparison between service types was not a primary question of the study but was notable in the qualitative data.

#### Recovery through relationships

The topic of how PARC services might enable personal recovery was probed in interviews. Carers responded that they were uncertain in their observation that PARC services enabled recovery. Only three qualitative survey comments specifically addressed the topic of recovery, perhaps unsurprising in regard to the inner experience of recovery for the person. However, some participants saw the importance of pursuing goals and identified that PARC services did support learning and recovery, including through the peer relationships. One carer thought more allied health staff could increase structured group activities to the group and individual support, to help residents with their individual goals/issues. Mainly, carers were focussed on the goal of overcoming a crisis, more than on the broader goal of recovery.

#### Limited access to PARC services

Though carers were overwhelmingly positive in describing PARC services, one consistent critical theme related to problematic experiences of accessing PARC services when needed. Because they valued PARC services, carers were frustrated and dismayed if they could not access PARC services as a crisis was developing, even if they sought help several times as this was occurring over months. Similarly, some carers were critical of PARC service stays being too short. It appeared that at some PARC services, the standard duration may be two weeks. In response to the survey prompt *if you could change one thing about PARC services* most participants referred to wanting either easier access or longer stays or more follow up on exit. One survey participant was dissatisfied, expressing concern that PARC service staff did not listen to the carer’s perspective. Many carers described consumers leaving PARC services before they were ready and felt that this was a constraint on the contribution of PARC services to recovery.

#### Little family involvement

At a fundamental level, several participants clearly indicated they wanted more communication with the PARC services team than was experienced. This contrasts with overall findings in the exit survey, that participants reported being listened too (84% agree/strongly agree) and receiving relevant and sufficient information (82% agree/strongly agree), but there were significant dissenting voices. Moving beyond pleasant communication and information sharing, family involvement in planning and care was lacking. In response to direct interview questions about *inclusion of families in planning and care*, several carers reflected they had little active involvement with PARC services, in accord with the item on the exit survey: *We have had an influence on how the service is run* (only 36% agreed). Not all participants were seeking such collaboration. However, participants who strongly identified with the role of carer explained they wanted to be involved more actively and in more depth. They were keen to continue with constructive strategies initiated in PARC services, to be “more of a resource” to the family member, and to learn about having positive family interactions. These carers felt under-prepared by PARC service teams to provide support when the persons returned home.

Overall, carers highly valued PARC services. In terms of critical comments, they mainly wanted easier access and more time with the particular residential settings, interactions, support and programs they had experienced at PARC services.

### Part 2: wellbeing of family/carers (associated with a PARC service stay)

This section begins with analysis of the quantitative data; the self-rated K10, Warwick, AQOL, considered important for family carer wellbeing associated with a consumer’s PARC service stay. Then themes from interview data and qualitative free-text items from the Exit Survey provide family members’ perspectives on the impact of PARC services for themselves. Together these data address the study question: *What was the impact of PARC service engagement on carers’ wellbeing and quality of life?*

#### Descriptive statistics for survey responses

**Table 4** presents the descriptive outcomes for all measures across the four phases of the study. Data for the Carer Exit survey was recorded only at time 2, *M* = 37.98, *SD* = 9.60, *N* = 50.

**Table 4:** Descriptive outcomes across four study phases.

Outcome		Phase				
		Time 1	Two weeks	Six months	12 months	Total
K10	*M*	19.42	18.86	17.92	17.63	18.43
	*SD*	7.68	6.75	6.10	6.63	6.77
	*N*	43	36	49	40	168
WEMWBS	*M*	49.54	50.73	50.10	52.03	50.44
	*SD*	8.37	7.94	7.96	8.92	8.27
	*N*	70	52	49	40	211
AQOL Mental	*M*	0.35	0.38	0.38	0.43	0.38
	*SD*	0.16	0.18	0.20	0.21	0.19
	*N*	64	51	49	40	204
AQOL Physical	*M*	0.67	0.68	0.67	0.68	0.67
	*SD*	0.23	0.24	0.24	0.25	0.24
	*N*	64	51	49	40	204
AQOL Utility	*M*	0.66	0.69	0.68	0.72	0.69
	*SD*	0.20	0.20	0.21	0.22	0.20
	*N*	61	51	49	40	201

Correlations among the outcome measures at each time point are shown in a **Supplementary Table**. In general, the descriptive results depicted in **Table 4** demonstrate change in the desired direction for all outcome measures: K10, and AQOL Mental ie higher quality of life, lower distress.

Growth curve analysis detected no significant trends higher than linear. Two outcome measures showed a significant linear trend in the expected direction: the K10, *F*(1, 52.32) = 4.96, *p* = .030, ${ƞ}_{p}^{2}$ = .09, 95% CI[<.01,.25], and AQOL Mental, *F*(1, 50.89) = 8.62, *p* = .005, ${ƞ}_{p}^{2}$ = .14, 95% CI[.01,.32]. The results approached significance for both WEMWBS, *F*(1, 58.48) = 3.24, *p* = .077, ${ƞ}_{p}^{2}$ = .05, 95% CI[<.01,.19], and AQOL Physical, *F*(1, 49.36) = 3.91, *p* = .053, ${ƞ}_{p}^{2}$ = .07, 95% CI[<.01,.23] and AQOL Utility was not significant, *F*(1, 80.47) = 0.78, *p* = .38, ${ƞ}_{p}^{2}$ = .01, 95% CI[<.01,.09]. All models found significant variability in intercepts at *p* <.001, but no significant variation in slopes, indicating that there was significant variability among participants at time 1, but no significant variability in the nature of participants’ growth trajectories. Significant variability across repeated measures was also found at *p* <.001 for all outcomes. This analysis examines trends and shows that carers started at significantly varying points on these measures. Descriptively we see that wellbeing and quality of life improved for carers over time, consolidating at the 12 month (time 4) point.

The participants’ responses regarding their wellbeing across the qualitative interviews and survey sources are reflected in the themes Relief, Respite and Learning for families as depicted in **Table 5**.

**Table 5:** Carer experience of PARC services & wellbeing - thematic structure and quotes.

Theme	Subtheme	Quotes
Relief, respite and learning for families	Relief and feeling good about PARC services	Eased my burden and showed us how to get help. (Survey) Well the staff were fantastic, they’re obviously used to people who need reassurance and who burst into tears at the slightest you know opportunity, and they were very, I just felt so glad to be able to trust her to their care (Interview)
		The thing about PARCS that it was just the most lovely environment, because it’s a lovely building, and all the staff were wonderful, and they were very, very warm and caring and calm, and I think that really helped [my daughter] – it certainly helped me. (Interview)
	Respite and trusting the team	It has also provided me with a break when I was at breaking point (Survey) I can only say from my heart a big thank you to PARC for giving my partner the opportunity of simply some time out and regrouping. Which gives us both positive outcomes (Survey)
		I felt completely at ease with my husband being cared for in this facility under the clinician [name] who was extremely attentive to my husband’s needs (Survey) I felt I had real help and I wasn’t alone any more, because of the existence of PARC as a service and more importantly, the people that work there (Survey)
	Learning from PARC services	Some of the things that the staff talked to me about, just little you know small conversations, just about the way [her daughter] or some of the other girls were feeling, or the way they reacted and what the staff did with them, you know it just was really helpful … I just felt I understood better (Interview).

#### Relief and feeling good about PARC services

All interviewees were asked explicitly about their own feelings and experiences of wellbeing. Several family members identified the time around PARC service stay as highly distressing for them, expressing their own emotions in self-deprecating terms, such as “people who need reassurance and who burst into tears.” One survey participant reflected on the powerful impact of PARC services at a point of personal extremity: “seriously on the edge of giving up on my own life”. Many carers reported very strong personal sense of relief associated with a PARC service stay.

#### Respite and trusting the team

Many interview and survey participants spoke of valuing the break and respite from responsibility that PARC services afforded them. The ability to experience respite was enabled when carers felt that they could trust the care at PARC services. In many instances carers named particular PARC service team members in whom they particularly placed their trust. Respite and relief for carers was intertwined in their experience with their sense of confidence in the care. Again, this experience was set against a background of struggles in help-seeking and distress carried by carers, about experiences of acute hospitalisation. Having trusted their family members to PARC services and teams, carers felt able to properly step back from caring responsibilities and regroup.

#### Learning from PARC services

Some carers were then also invested in learning more about mental health and wellbeing, for themselves and for their family member. They appreciated the opportunity to learn in conversation with PARC service staff, or by being present when staff and consumers were interacting and engaged in groups. As noted in an earlier section, many carers wanted more opportunities to learn from the PARC service team about the best ways to support their family members.

In general, the personal experience and impact of PARC services for family members, carers and supporters was emotionally powerful, though often understated. They touched on their sense of “burden” in a “difficult time”. Carers spoke of themselves as being at “breaking point” and PARC service involvement as “a lifesaver”, and “critical” for their family member. Finding PARC services to be a trustworthy and safe place, they were able to experience “much needed relief” for themselves. From that point, some carers were ready and keen to engage and learn.

## Discussion

Recovery oriented practice and service delivery have previously been relatively silent regarding the role of carers and other informal supporters, and the impact of recovery oriented service delivery and new and emerging models of care on carers. This is despite evidence that most consumers experiencing mental health problems have ongoing relationships with their families, that families and supporters often need support for themselves and want to be involved in the care of their loved one (1). This project deliberately included carer perspectives and also anticipated that a stay at a PARC service may have an impact on the wellbeing of that person’s carers.

The findings confirm that carers have important perspectives on PARC services. It appears that PARC services can support carers who are distressed and experiencing a family crisis in the context of the person they care for requiring an increased level of care. For many carers a PARC service admission follows experiencing their loved one having an inpatient admission – described as step down admissions - and carers in this study frequently positively compare PARC services to inpatient units and see PARC services as a valued alternative, as have consumers (19, 33). While carers conveyed little to no scope to influence the service provision, they were highly positive both about its contribution to them and the consumer and their wish to access PARC services into the future.

Further, the findings highlight contributions of a PARC service stay to wellbeing of the carers. Quantitative data showed the participants quality of life and experience of distress improved across time and qualitative data (from open ended survey responses and a small number of interviews) conveyed that the experience of respite from worry about the person’s safety and from caring roles were fundamental to this improvement (34). When families spoke of PARC services as safe and supportive, their gratitude was heightened by contrast with other experiences of feeling overwhelmed and lacking support and being deeply concerned for the suffering and situation of their family member, whether at home or in hospital. This concern is echoed in studies of family/carer distress through mental health crisis care (4). Carers in Australia (35) and internationally (36) report that their own recovery and sense of respite is constrained when a family member is in hospital care, because of distress and concern about adverse consumer experiences in these settings. In an interview study carers also report needing and not receiving support for trauma associated with crises and crisis responses by police and community teams and subsequently they avoided help seeking for themselves and the consumer (37). The carer experiences of PARC contrasts with such adverse experiences.

However, some of the features of previous research exploring family experiences is repeated here. Families and carers in this study, despite their generally very positive feedback, expressed that they did not have the amount of information about the services as they would have preferred, both prior and during the stay. They also commonly experienced a lack of engagement and involvement in the care of their loved one. This aligns with Harvey et al. (15)’s findings regarding a lack of consistent involvement of carers by PARC services, despite this being a goal. Also, for some carers and families PARC services were a limited resource – both in in the ability to access the service and in relation to the length of stay. While access is less than desired for any residential support through crisis (38), a clear message from carers is heightened by their un-alloyed positive experience of PARC services. Interview methods yielded these nuanced findings; satisfaction surveys (such as the exit survey) are criticised as a measure of experiences and these are best learned from qualitative approaches (39), affirming the value of this mixed method approach to the question.

Our PARC services program of research found evidence that PARC services are associated with many consumers reporting a more positive trajectory in relation to their service use, recovery and wellbeing (40). These findings suggest this may be similarly the case for carers. In relation to our outcome measures, while there was significant variability at time 1, we see a relatively positive trajectory for the participants following contact with a PARC service through improvements in quality of life and reduced distress over time. This contrasts with findings by Poon et al. (6) that did not see a change over time on similar measures. While a direct comparison needs to consider that Poon et al. (6) did not undertake time one assessments at a time of crisis, which it could be argued was a stronger feature of this sample, we do see that the trajectory families and carers went on shares similarities with consumers of PARC services. The qualitative data also supports these outcomes. Although only collected at time 2, the exit survey does suggest that carers and families achieved personal benefits from a PARC service stay – such as “a life saver” for all family members and being relieved from high levels of distress, a finding in line with consumer and carer aspirations for crisis care (38).

However, the findings suggest that overall there was only modest engagement with carers in general –and some scope to increase communication with carers. This is consistent with the potential that others have identified for recovery-oriented practice to take a more relational and family focus (41). This suggests that personal recovery is linked to interpersonal relationships with others. As Rhys Price-Robertson et al. (41) suggest, people with lived experience normally adopt, or at least aspire to adopt, numerous different relational identities – spouses, lovers, friends, children, parents, siblings, aunts and uncles, grandparents, carers of loved ones themselves – all of which entail complex networks of interdependence. (p. 117). In this study, all the participating carers were nominated by the consumer, reflecting that they are an important person in the consumer’s life and potentially carry an ongoing caring and support role (13), and yet carer participants identified patchy opportunities for involvement in PARC service work, to support the person’s own recovery.

PARC services may be missing opportunities for developing the wellbeing of whole families, enhancing family interaction and enabling family or relational recovery (22). Active engagement with families and others in caring roles, including attentive listening to their needs (42), would require development of the current model of care, necessitating careful attention to information sharing with regard to consumer autonomy and confidentiality. Hence, recovery-oriented practice could be extended to be less individualistic and more consistently inclusive of carers and family members who might then be better informed, supported and included.

Finally, a valued role of PARC services for carers is respite and this appears to assist carer wellbeing (34). Currently, the description of these services as ‘sub-acute’, which suggests they are not providing urgently required care, is dissonant with common participants’ reports of experiencing crises and intense distress at the point of a PARC service admission. There may be potential for PARC services to do more in that space, such as through more opportunities for planned PARC service stays, although this may also depend on the expansion of the availability of PARC services.

### Limitations

An important limitation in this study is the timing of the time 1 survey completion. This relied on consumers nominating a carer or family member to participate in the study and also contributed to delays in then making contact and recruiting carers (43). Hence time 1 may have occurred well into the consumer’s PARC service stay so may not fully reflect a “baseline” or at least the time when participants were most in need of PARC services support for themselves and the person they care for or loved one. Another limitation was the high rate of missing data at the later timepoints. This was accommodated for in power considerations for our analysis. Also the sample did not capture the experience of a significant minority of carers with language other than English (LOTE). This is a limitation, as carers in LOTE communities are known to have specific mental health service needs (44) and poorer experience of health services in general. A strength of the qualitative interviews was that they were conducted by a carer researcher, to encourage open and safe communication, and the sample was drawn from 6 different services, adding credibility to the discussion of PARC services as a model of care. But the study included only a small number of interviews that were brief and by telephone. We do not claim that the modest qualitative interview sample achieved saturation but they did provide complementary insights to the qualitative and qualitative data in the survey. Together the mixed methods data provides a valuable account of carer experiences. In terms of the quantitative data analysis, it is important to note the high level of missing data for some measures at certain time points. Even though the missing data were found to be missing at random, which supported the use of full information maximum likelihood estimation in our modelling, imputation was considered inappropriate. This level of missing data should be considered a limitation, despite appropriate statistical attempts to account for it. There is the possibility that participants who did not provide data at all time points were qualitatively distinct from those who did. Further, the different timing associated with the administration of time 1 surveys complicates interpretation of the linear mixed model growth curves by impacting the results of the random intercepts. Finally, it is important to note that the effect sizes reported for the inferential results were generally in the small to moderate range, according to convention; therefore, the clinical and practical significance of these findings needs to be considered in the context of this modest magnitude of effect.

## Conclusion

We found that carers generally considered PARC services to be a positive environment for the person they cared for (or loved one) to receive treatment and support. We also found promising evidence in support of the association between PARC services and improvements in the quality of life and wellbeing of carers. However, PARC services have the potential in future to further enhance the engagement of carers in service development and improvement. This could be achieved through more consistent efforts to receive feedback, such as via an exit survey, and emphasis on experience based co-design. This study contributes to a growing body of evidence about how highly valued recovery oriented sub acute residential services are for services users. It also supports the potential for more emphasis on relational recovery to be a feature of the PARC service model, to acknowledge the potential for carers to benefit from PARC services as well as enabling them to contribute to consumer recovery.

## Data Availability

The raw data supporting the conclusions of this article will be made available by the authors, without undue reservation.
