# Supplementary material for: Experiences and wellbeing of family members and carers, regarding PARCS across Victoria
Source: Front Psychiatry. 2026 Jun 16;17:1777053. doi: 10.3389/fpsyt.2026.1777053 (PMC13315014; doi:10.3389/fpsyt.2026.1777053)
Supplement: Supplementary file 1 [file DataSheet1.pdf]

Supplementary Table: *Correlations Among Outcome Measures at Four Time Periods*

Phase	Outcome		K10	WEMWBS	AQOL Mental	AQOL Physical	AQOL Utility
Time 1	K10	*r*	-	-.43	-.74	-.61	-.81
		p	-	.004	<.001	<.001	<.001
		N	-	43	38	39	36
	WEMWBS	*r*		-	.77	.45	.70
		p		-	<.001	<.001	<.001
		N		-	64	64	61
	AQOL Mental	*r*			-	.62	.94
		p			-	<.001	<.001
		N			-	61	61
	AQOL Physical	*r*				-	.82
		p				-	<.001
		N				-	61
Time 2 Two Weeks	K10	*r*	-	-.59	-.65	-.42	-.66
		p	-	<.001	<.001	.010	<.001
		N	-	36	36	36	36
	WEMWBS	*r*		-	.73	.27	.62
		p		-	<.001	.054	<.001
		N		-	51	51	51
	AQOL Mental	*r*			-	.53	.92
		p			-	<.001	<.001
		N			-	51	51
	AQOL Physical	*r*				-	.78
		p				-	<.001
		N				-	51
Time 3 6 Months	K10	*r*	-	-.63	-.75	-.52	-.79
		p	-	<.001	<.001	<.001	<.001
		N	-	49	49	49	49
	WEMWBS	*r*		-	.81	.50	.73
		p		-	<.001	<.001	<.001
		N		-	49	49	49
	AQOL Mental	*r*			-	.59	.91
		p			-	<.001	<.001
		N			-	49	49
	AQOL Physical	*r*				-	.81
		p				-	<.001
		N				-	49
Time 4 12 Months	K10	*r*	-	-.69	-.75	-.72	-.85
		p	-	<.001	<.001	<.001	<.001
		N	-	40	40	40	40
	WEMWBS	*r*		-	.82	.65	.84
		p		-	<.001	<.001	<.001
		N		-	40	40	40
	AQOL Mental	*r*			-	.70	.92
		p			-	<.001	<.001
		N			-	40	40
	AQOL Physical	*r*				-	.86
		p				-	<.001
		N				-	40
